# Predicting the Use of Public Transportation: A Case Study from Putrajaya, Malaysia

**DOI:** 10.1155/2014/784145

**Published:** 2014-07-09

**Authors:** Muhamad Nazri Borhan, Deprizon Syamsunur, Norliza Mohd Akhir, Muhamad Razuhanafi Mat Yazid, Amiruddin Ismail, Riza Atiq Rahmat

**Affiliations:** ^1^Department of Civil and Structural Engineering, Universiti Kebangsaan Malaysia (UKM), 43600 Bangi, Selangor, Malaysia; ^2^Sustainable Urban Transport Research Centre (SUTRA), Universiti Kebangsaan Malaysia (UKM), 43600 Bangi, Selangor, Malaysia; ^3^School of Civil Engineering, Linton University College, Batu 12, Mantin, 71700 Mantin, Negeri Sembilan, Malaysia

## Abstract

Putrajaya is a new federal administrative capital of Malaysia which has been set to achieve a 70% share of all travels by public transport in the city area. However, the current modal split between the public transport and private transport is 15 : 85. In order to understand travelers' willingness to use the public transport, a conceptual model has been developed to determine the factors that affect them to use the public transport instead of travelling in their own cars. Various variables such as service quality, environmental impact, attitude, and behavior intention were analyzed and tested using structural equation model (SEM). Results indicate that the service quality and attitude are found to have positive effects on the behavioral intention of taking the public transport. Other than this, this study also shows that the service quality and environmental impact have some positive influences on the attitude to using the public transport. However, environmental impact has no significant, positive, and direct effect on behavioral intention. The results of this study demonstrate that the model that was developed is useful in predicting the public transport and it could provide a more complete understanding of behavioral intention towards public transport use.

## 1. Introduction

The increasingly active car use in and around the cities in industrialized countries has led to the increased accessibility problems, as reflected in traffic jams and parking problems. Besides road congestions, private cars also cause serious problems such as CO_2_ pollution, global warming, and noise [1–5]. The private car is one of the major modes of personal transport in Malaysia, mainly because it is affordable and more reliable than the other transport available. The government has given its full support to this local car manufacturing company, through a variety of policies which seek to cover this company. Through this support, the imported vehicles in Malaysia have been charged as much as 140 to 300 percent excise duty to ensure that the local car makers are competitive in terms of price compared to imported cars [6]. Thus, about 47.8% of the registered vehicles in the country are private cars, whose numbers had increased tremendously from 458,294 in 2006 to 628,239 in 2012 [7].

In recent years, many studies have been conducted to reveal the factors that can reduce private transport use and increase the number of ridership of the public transport in the city area. Among the many factors examined, much of the literature had emphasized on the importance of service quality factors in encouraging people to switch to the public transport. Service quality has been dominating the construct towards public transport studies [8–11]. Besides, variables which are related to environmental concern have also been found to be associated with the public transportation use or car use in previous studies [12–15]. Hence, consistent with the issues of global warming, a better understanding of the influence of more environmentally friendly modes of transport such as the public transport, which produces less emission compared with private vehicles, is particularly important. Apart from the service quality and environmental impact, the role of attitude is also seen as contributing to the shift made by people from private vehicles to the public transport. Based on the theory of planned behavior (TPB), attitude refers to a person's overall evaluation of the behavior of either the favorableness or the unfavorableness towards performing a behavior [16, 17]. Therefore, customer attitude is closely linked with the perceived service quality provided by the public transport and the perceived environmental impact caused by private vehicles. People who perceive the good quality of public transit service and perceived environmental impact caused by private vehicles are thus more likely to have a higher attitude towards the intention to use the public transport.

The aim of this paper is to provide a basic framework and a set of strategies to improve public transport services that are readily available in Putrajaya by changing the behavior of private vehicle users from using private vehicles to public transport. Specifically, the aim is to develop a relationship model based on the conceptual framework that incorporates the variables that affect the behavioral intention of private vehicle users to use public transport and to explore their effects on behavioral intentions.

## 2. Research Area

Putrajaya is a new administrative capital of Malaysia located some 25 kilometres south of Kuala Lumpur. The need for a new capital city was seen inevitable because of the severely congested conditions and due to land limitations in Kuala Lumpur. It is also in line with the aim of having a new and well-planned capital city that reflects the Malaysian identity, befitting the country's aspiration to become a fully developed nation in 2020. A total of 25 ministries and 51 government offices have moved to Putrajaya and they will provide 254,000 job opportunities. Putrajaya has been set to achieve a 70% share of all travels by the public transport in the city area. However, this goal appears to be very difficult to achieve because in the current situation this goal entails a reversal of the current modal split of 15 : 85 between the public and private transports [18, 19]. The transport action plan study has revealed that the average vehicle occupancy for private cars commuting to Putrajaya daily was around 1.69 persons. The field investigations also show that the average vehicle occupancy for a public bus was around 25 persons compared with its legal load capacity of 44, indicating that it has failed to attract the public [20].

## 3. Theoretical Background and Hypotheses

### 3.1. Service Quality

Service quality is a measure of how well the service level that is delivered matches customers' expectations, while delivering quality service means conforming to customer expectations on a consistent basis [9, 11]. To increase the usage of the public transport, both the operators and the authorities need to understand how consumers evaluate the quality of services provided [21].

Joewono and Kubota [9] measure the service quality of Indonesian paratransit systems using nine factors with 54 attributes. The results show that the service quality has positive effects on both the overall satisfaction and customer loyalty, and the overall satisfaction has a positive impact on customer loyalty. Meanwhile, Lai and Chen [10] have measured the service quality of the mass rapid transit (MRT) system in Kaohsiung city, Taiwan. They have asked the MRT users about the service quality which is divided into two factors, namely, core service factor and physical environment factor. The core service factor consists of the items as follows: service frequency, general information provision, network coverage, service provision hours, prices of tickets, service frequency, complaint dealing, ticket selling network, train information provision, and personnel behavior. Meanwhile, there are eight items under physical environment factor which are facility and vehicle cleanliness, vehicle safety, safety at terminals and stops, vehicle stability, conditions at terminals and stops, and onboard information provision. That study reveals the aspects of service quality which have significant effects on behavioral intentions mediated by perceived value, satisfaction, and involvement.

In the present study, the service quality attributes are mainly adapted from Lai and Chen [10] and amended according to the specific service characteristics of the Putrajaya Public Transport System.

### 3.2. Environmental Impact

The present study also examines the variable-related environmental concern that has been found to be associated with the public transportation use or car use in previous studies. Himanen et al. [13] have suggested that by implementing transport policies such as decreasing the vehicle mile travelled, auto production, and ownership and increasing the use of technological measures for cleaning exhaust gases, environmental quality/sustainability can be improved. The finding is also supported by a research by Steg and Vlek [15] which investigates the role of awareness of and the perceived responsibility for the problems caused by car use in a study of car use in the Netherlands. Car users with greater awareness of the problems tend to feel more responsible for the problems caused by car use, and, subsequently, they use their cars less. A study of the determinants of transportation mode choice in Sweden [12] also found that environmental concern and a perceived causal link between car use and environmental problems predicted the intention to reduce car use. The present study explores the generalizability of these findings. In addition, recently concluded research [14] proves that the satisfaction has significant effects on customer's loyalty to use public transport mediated by environmental concern. The goals are to encourage and improve the more environmentally friendly modes to make efficient use of the existing public transport. Our study explores the role of environmental values in relation to other values as a predictor of public transportation use.

### 3.3. Attitudes

Attitude refers to a relatively persistent and consistent behavioral inclination of individuals based on their recognition and based on the likes and dislikes of people, event objects, and the environment [22]. Lippa [23] considered attitude as a kind of evaluative response (like or dislike) towards a particular object. He emphasized it as an intervening variable in social psychology research and a hypothetical construct that can be inferred but cannot be directly observed. As is the case with the travel behaviour, it is not influenced by the service level of the transport system but is influenced by psychological factors such as perceptions, attitudes, and habits [17, 24]. According to Ajzen and Fishbein [25], attitudes towards the behavior reflect the overall evaluation of performing the behavior by the individual in the theory of planned behavior (TPB). Attitudes are based on the expectancy beliefs about the likelihood that behavior will result in particular consequences and on the evaluations of the desirability of those consequences [25]. Having applied this to the study, it is suggested that service quality and environmental concern will be related via the attitudes towards the public transport. In addition to our proposed model, there are other alternative models that will be derived. For example, service quality and environmental concern could also affect behavioral intention directly.

**Figure 1:**
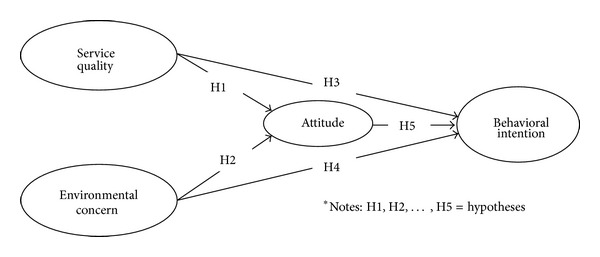
The conceptual model.

Based on the review of the literature, the current study proposes the conceptual model shown in Figure 1. The hypotheses are stated as follows.

H1: service quality is positively related to the intention towards public transport use via attitude.
H2: environmental concern is positively related to the intention towards public transport use via attitude.
H3: service quality is positively related to the intention to use public transport.
H4: environmental concern is positively related to the intention to use public transport.
H5: attitude is positively related to the intention to use public transport.

## 4. Methods

### 4.1. Sample and Data Collection

Data collection from the respondents was conducted through questionnaires distributed to government departments and ministries in Putrajaya. The majority of the respondents who participated in this study are working in the areas of administration and management at the government departments and ministries. In this study, we had used a stratified sampling method in the approach to obtain a sample of respondents. A total of 500 self-administered questionnaires were distributed throughout the data collection period. Only 304 questionnaires were returned in which 14 forms were rejected as invalid; only 290 questionnaires received were considered for further action (58% response rate). A minimum sample size of 200 was needed to reduce biases to an acceptable level for any type of SEM estimation [26–29]. There are several studies related to the SEM that use less than 300 samples, such as on seatbelt use (*N* = 277) [30], motorcyclists' intention to speed (*N* = 110) [31], prediction of the electronic toll collection service adoption (*N* = 255) [32], users' satisfaction on urban public transport services (*N* = 140) [33], and influences of paratransit as a feeder of the mass transit system (*N* = 125) [34]. Specific demographic information is shown in Table 1.

As shown in Table 1, the majority of respondents (66.6%) are in the age group of 30 and below and 56.6% are females. This indicates the participation of women in the employment sector at present has increased substantially, in which women also contribute to using cars to go to work and also to send and pick up children at the child care center [35, 36]. Furthermore, younger people are more likely to use their own vehicles [37]. In addition, most respondents are highly educated with 66.2% of them having attained university diplomas, university degrees, or postgraduate degrees.

### 4.2. Measurement Development

The questionnaires were designed to collect the required information from the respondents for research purposes. The questionnaire was divided into tree main aspects. The subjects contained in the questionnaire are as follows:

- Part A: background of respondents.
- Part B: information on respondents' travel.
- Part C: information based on the conceptual model.

In Part C, the questionnaire examined service quality (9 items; example of item is as shown in Table 2), environment (two items; example of item is “Using the car contributes to environmental pollution”), attitude (three items; example of item is “Using the public transport is a good idea”), and behavioral intentions (two items: “I will try to use the public transport” and “I intend to use the public transport”).

Before the data collection activities were conducted, a preliminary test was conducted to ensure that the questions in the questionnaire form are easy for the respondents to understand and answer. According to Cooper and Schindler [38], a preliminary test sought to determine if there was a mistake or an error in the design of instrumentation to enable researchers to rectify them before adopting them in the actual study. A preliminary testing of 50 samples of respondents from among the postgraduate students taking courses in transportation engineering at Universiti Kebangsaan Malaysia (the National University of Malaysia) was done to get the information and feedback on the questionnaire. In preliminary tests conducted, the overall respondents had understood the questions presented in the questionnaire. Apart from the demographic and travel behavior information, which were measured by the categorical scales, the items of all the other constructs were measured using a seven-point Likert scale with 1 = Strongly Disagree, 7 = Strongly Agree, and 4 = neither agree nor disagree. According to Finstad [39], the 7-point Likert scale provides a more accurate measure of a participant's true evaluation and it is more appropriate for electronically distributed and otherwise unsupervised usability questionnaires.

### 4.3. Data Analysis

In the present study, we had conducted the data analysis in two stages. First of all, exploratory factor analyses using the principal component with the VARIMAX rotation technique were performed to examine construct dimensionalities. VARIMAX, which was developed by Kaiser [40], is undoubtedly the most popular rotation method by far. For VARIMAX, a simple solution means that each factor has a small number of large loadings and a large number of zero (or small) loadings. This simplifies the interpretation because, after a VARIMAX rotation, each original variable tends to be associated with one (or a small number) of factors, and each factor represents only a small number of variables. In addition, the factors can often be interpreted from the opposition of few variables with positive loadings to few variables with negative loadings. The factor was retained only if it had an eigenvalue, that is, the amount of variance accounted for a factor, which is greater than 1.0. The items under each factor were retained only if they had factor loadings greater than 0.5. To assess the reliability of measures, Cronbach's Alpha was calculated to examine the reliability of variables retained in each factor, and coefficients greater than 0.7 were considered acceptable, indicating a good construct reliability [41]. On that basis, the relationships of intention, attitude, subjective norms, perceived behavior control, situational factor, and trust were empirically tested using the SEM in the second stage by using AMOS 19.

## 5. Results and Discussion

### 5.1. Dimensionality of Service Quality

This study employs a multiattribute approach to measure public transport service quality. The results from the principal component factor analysis, a unidimensional solution with nine service quality items, explain 66.9% of the total variance. The quality of service factor has an eigenvalue of 6.025 and reliability value (*α*) of 0.911. The factor loading value obtained for all nine items demonstrates values that exceed the minimum value of 0.5 as shown in Table 2. Besides, the KMO measure of the sampling adequacy is 0.911, which is above the acceptable value of 0.6. Furthermore, Bartlett's test of sphericity is significant; thus, the hypothesis that the intercorrelation matrix involving these nine items is an identity matrix is rejected. Thus, from the perspective of Bartlett's test, the factor analysis is regarded as feasible.

### 5.2. Measurement Model

During the development of the structural equation model (SEM) in this study, we have followed two methods (measurement and structural models) proposed and recommended by Anderson and Garbing [42] and Hair et al. [43] to assess the construct validity, the model fit, and the hypotheses. Anderson and Garbing [42] proposed a two-step approach (measurement and structural models) that has several advantages which are directly related to the structural model. In addition, the analysis of the two-step approach provided complete and comprehensive data to estimate the validity of the construct, to test the hypotheses, and to evaluate whether the structural model fits the theoretical model and is acceptable. The Confirmatory factor analysis (CFA) was used to estimate the convergent and discriminant validity using the AMOS 19.0 program. The overall goodness-of-fit indices of the CFA measurement model indicate a satisfactory fit of the measurement model with Chi-square per degree of freedom (*χ* ^2^/degree of freedom) = 2.54, GFI = 0.93, NFI = 0.95, TLI = 0.95, CFI = 0.96, and RMSEA = 0.07.

Convergent validity ensures that the item measures or the indicators have a loading factor that shows that they converge at one point. The literature has suggested three criteria to assess convergent validity [43, 44].

1. The loading factor for each item must be significant and its value must exceed 0.5.
2. The construct reliability for each construct should exceed 0.50.
3. The average variance extracted (AVE) for each construct should exceed 0.50.

Table 3 shows that all the CFA item values had exceeded 0.5 and were significant at *P* = 0.01 except for one item from the situational factor, which had the loading factor of less than 0.5; that item was dropped from further analysis. The composite reliability analysis shows that all constructs had a value over the recommended threshold value of 0.5 [43]. The composite reliabilities of the constructs ranged from 0.59 to 0.90. Furthermore, the AVE ranged from 0.50 to 0.73, also above the acceptable value of 0.5. Apart from that, the Cronbach alpha value obtained for all constructs demonstrates that the value exceeded the minimum value of 0.7 as recommended by Nunally [41]. As shown in Table 4, all variables were significantly correlated with behavioral intention. Attitude (*r* = .58) had a strong relationship with behavior intention followed by service quality (*r* = .37) and environmental impact (*r* = .27). Service quality (*r* = .53) had a strong relationship with attitude, followed by environmental impact (*r* = .29). Meanwhile, the variable mean, standard deviation, and correlation are shown in Table 4. All constructs were significantly correlated with behavior intention.

### 5.3. Structural Model

**Figure 2:**
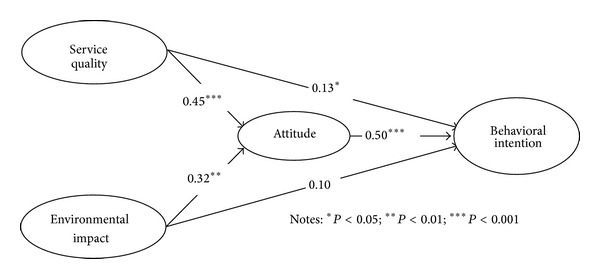
Result of the research model.

Using the measurement model that has met all the conditions for convergent and discriminant validity, a complete structural model was developed to test, investigate, and evaluate the significance and strength of each path in the model. The result of the analysis of the complete structural model towards public transport use is shown in Figure 2. The results have shown that most goodness-of-fit indices are within acceptable levels (*χ* ^2^/degree of freedom = 496.84/191 = 2.60, RMSEA = 0.074, NFI = 0.90, TLI = 0.92, CFI = 0.93, GFI = 0.87, and AGFI = 0.85).

Figure 2 shows the details about the parameter estimates for the model and Table 5 reports the results of the hypothesis tests. On the whole, four out of five hypotheses are supported. Service quality (*β* = 0.45, *P* < 0.001) and environmental impact (*β* = 0.32, *P* < 0.01) positively influence the attitude. Therefore, hypotheses 1-2 can be accepted. As hypothesized, service quality has significant positive effects on behavioral intention (*β* = 0.13, *P* < 0.05), thus supporting H3. However, environmental impact has no significant positive direct effect on behavioral intention; thus, H4 is not acceptable. Finally, attitude has a significantly positive effect on behavioral intentions (*β* = 0.50, *P* < 0.001), which is supporting hypothesis 5.

This study reveals the aspects of service quality that have a significant potential to improve the car users' attitude in order to attract new consumers to use public transport and to maintain existing customers. The priorities for improving the quality of service can be seen based on the regression weights of the quality of service towards attitude in Figure 2. According to our results, it is found that factors such as service reliability, safety (at bus stop/terminal and onboard), customer service, and provision of adequate information of bus route are directly significant towards behavior intentions and indirect significant influences mediated by attitudes. This explains that the increasing service quality of public transport will increase the positive attitudes toward behavior intention to use public transport. The responsible agencies such as the government and Putrajaya Corporation should be involved in improving the quality of service to enhance consumer attitude and intention towards public transport in the future. As reported by M. A. R. Nor and M. N. G. Nor [20], the public bus system in Putrajaya is not popular among the public because the service is unreliable, not punctual and the travelling times are longer. Indirectly, the poor quality of service experienced by users will have an impact on customers' overall satisfaction on public transport and this will increase the negative attitude to the intention to use public transport. Thus, the service quality can affect the public's intention to use the public transport through attitude. According to our results, service attributes such as safety (onboard and at the bus stops or terminal), customer service, and information related to the services provided such as bus frequency, number, and route have significant influences on car users' behavioral intentions. These findings can provide useful information for the public transport providers in the efforts to prioritize the important service attributes and ensure that the service quality is able to meet consumer expectations.

Meanwhile, the environmental impact has no significant impact on the behavior intention to use public transport. This shows that directly, people are not worried about the effects of pollution produced by their own vehicles on the environment. This causes them to not have the intention to use public transport to reduce the environmental impact. However, the environmental impact is seen to affect the intention indirectly mediated by attitude variables. This shows that more and more private motorists are concerned about the consequences of environmental problems produced by their vehicles; then, they will adopt a more positive attitude and develop the intention to use public transport. If customer satisfaction can be met, such as providing a good service, this will help realize the move to achieve a sustainable transport system by changing the mode of transport to save the environment. The results have also suggested that the need of both government and nongovernment organisations (NGO) to promote an awareness campaign to protect the environment from pollution caused by the use of private vehicles to influence car users' attitude to use public transport should not be negligible.

Table 6 has shown the effects (i.e., direct, indirect, and total) of all relationships. First of all, the direct effects of service quality and environmental impact on attitude are 0.45 and 0.32, respectively. This shows that the service quality has the greatest effect compared to the effect of environment towards attitude. Secondly, the direct effects of service quality and attitude on behavioral intentions are identified, where the result shows that the latter (0.50) is greater than the former (0.13), while there is no direct effect of the environmental impact on behavioral intentions found. With respect to the indirect effects, thirdly, the effect of service quality on behavioral intentions mediated by attitude is 0.23, and this brings the total effect of service quality on behavioral intention 0.36. Additionally, the indirect effect of the environmental impact on behavioral intentions mediated by attitude is 0.16, hence resulting in a total effect of 0.16.

The quality of service is the strongest predictor of attitudes towards the behavioral intentions and it has some practical implications for local authorities. First of all, in order to promote attitudes in favour of using the public transport, it is important to focus on messages that emphasize individual advantages of using the public transport. The service provider should monitor whether or not the use of the public transport indeed provides the expected individual advantages. The existing public transport system in Putrajaya lacks the quality and availability and also, it takes longer travelling times [18, 20]. These factors can be seen as pressures or constraints on time for the consumers. To ensure successful implementation of public transport in Putrajaya, those factors should be taken into account especially to improve the quality of services. For example, by increasing the number of buses, it will also increase the frequency of buses and reduce bus travel time. This is because, for those who use the car to work, they do not want to wait for the bus because the bus tends to arrive inconsistently. Besides, they also feel that the bus will unnecessarily consume more of their travel time to get to work. For the long term period planning, the policy-related financial disincentives such as congestion toll pricing or cordon charging should be implemented. As a policy tool, such a steep increase appears to be unrealistic for immediate implementation because it is very likely to be politically unpopular. However, increases in penalties for private vehicle travel may be introduced in stages over a longer period of time along with the improvements in the public transport system.

## 6. Conclusion

This study has highlighted the major factors in affecting the travel mode choice by adopting the variables by incorporating those variables into the conceptual model in the present study. These factors are grouped and modeled into 3 categories in AMOS 19 software where they were analyzed for assessing the effect on behavioral intentions. Major conclusions drawn from this study are as follows.

- The sample size of 290 involved in the study was adequate and validated through the overall goodness-of-fit indices for measurement and structural models.
- The most significant category of influence factors affecting the behavioral intention of private vehicle users to use public transport is attitude and this is followed by service quality.
- More research in future should be conducted to find out the effect of attributes related to financial disincentives such as toll pricing congestion or cordon charging to encourage people to use the public transport in Putrajaya.

## Figures and Tables

**Table 1:** Demographic information.

	Frequency	Percentage
Age		
30 and below	193	66.6
31–40	68	23.4
40–49	14	4.8
50 and above	15	5.2
Total	**290**	**100**
Gender		
Male	126	43.4
Female	164	56.6
Total	**290**	**100**
Education		
High school	98	33.8
Diploma	101	34.8
University	67	23.1
Postgraduate degree	24	8.3
Total	**290**	**100**

**Table 2:** Factor analysis of service quality.

	Items	Factor loading	Eigenvalue	Variance explained (%)	Cronbach's *α*
QS1	Service reliability	0.792	6.025	66.946	0.937
QS2	Safety at bus stops/terminal	0.853			
QS3	Onboard safety	0.898			
QS4	Customer service	0.905			
QS5	Information	0.886			
QS6	Vehicle cleanliness	0.832			
QS7	Ticket selling network	0.768			
QS8	Luggage storage area	0.813			
QS9	Special bus lane	0.810			

Kaiser-Meyer-Olkin measure of sampling adequacy = 0.911.					
Bartlett's test of sphericity = 2564.544 (d.f. = 136, *P* = 0.000).					

**Table 3:** Standardized loadings and reliability.

Constructs and items	Standardized loading	Contruct realibility	AVE	Cronbach's *α*
Service quality (SQ)		0.881	0.50	0.94
SQ1	0.547 (*t* = 10.09)			
SQ2	0.888 (*t* = 19.83)			
SQ3	0.984 (*t* = 23.98)			
SQ4	0.917 (*t* = 27.75)			
SQ5	0.888 (*t* = 25.76)			
SQ6	0.698 (*t* = 14.33)			
SQ7	0.649 (*t* = 12.93)			
SQ8	0.523 (*t* = 9.57)			
SQ9	0.630 (*t* = 11.95)			
Enviroment (ENV)		0.763	0.62	0.82
ENV1	0.875 (*t* = 10.95)			
ENV2	0.793 (*t* = 10.45)			
Attitude (ATT)		0.905	0.76	0.91
ATT3	0.924 (*t* = 26.75)			
ATT4	0.943 (*t* = 21.57)			
ATT5	0.809 (*t* = 19.76)			
Behavioral intention (B.INT)		0.797	0.67	0.84
B.INT1	0.895 (*t* = 12.45)			
B.INT2	0.811 (*t* = 11.67)			

**Table 4:** Means, standard deviations, and correlations.

	Mean	STD	SQ	ENV	ATT	B.INT
SQ	5.60	1.23	1			
ENV	5.05	1.24	0.29	1		
ATT	4.98	1.40	0.53	0.29	1	
B.INT	4.56	1.55	0.27	0.37	0.58	1

Note: all correlations were significant at *P* < .01.

**Table 5:** Hypothesis tests.

Path relationships	Structural coefficients	*t*-value	Test result
H1: quality of service → attitude	0.45	4.64***	Support
H2: environment → attitude	0.32	3.90**	Support
H3: quality of service → behavioral intention	0.13	2.18*	Support
H4: environment → behavioral intention	0.10	1.31	Reject
H5: attitude → behavioral intention	0.50	6.45***	Support

*P < 0.05; **P < 0.01; ***P < 0.001.

**Table 6:** Direct, indirect, and total effects of relationships.

Path relationships	Direct effect	Indirect effect	Total effect
Attitude → behavioral intention	0.50	—	0.50
Environment → attitude	0.32	—	0.32
Quality of service → attitude	0.45	—	0.45
Quality of service → behavioral intention	0.13	0.23	0.36
Environment → behavioral intention	—	0.16	0.16
